# Awareness of human papilloma virus and cervical cancer prevention among Cypriot female healthcare workers

**DOI:** 10.3332/ecancer.2019.978

**Published:** 2019-11-20

**Authors:** Andria Christodoulou, Jirayr Ajzajian, Dejun Su, Hongmei Wang, Zoe Roupa, Paraskevi A Farazi

**Affiliations:** 1 University of Nicosia, 46 Makedonitissas Avenue, Nicosia 2417, Cyprus; 2 Cyprus Institute for Environmental and Public Health, Cyprus University of Technology, Limassol 3036, Cyprus; 3 Department of Health Promotion, College of Public Health, University of Nebraska Medical Center, Omaha, NE 68198, USA; 4 Department of Health Services Research and Administration, College of Public Health, University of Nebraska Medical Center, Omaha, NE 68198, USA; 5 Department of Epidemiology, College of Public Health, University of Nebraska Medical Center, 984395 Nebraska Medical Center, Omaha, NE 68198, USA

**Keywords:** human papilloma virus, cervical cancer prevention, awareness, healthcare professionals, Cyprus

## Abstract

**Background:**

Cervical cancer incidence varies around the world with the highest rates in Eastern Africa and the lowest rates in Western Asia. In Cyprus, a small Mediterranean island, cervical cancer incidence was 6.4 per 100,000 in 2013. HPV is an established risk factor for cervical cancer with HPV-16 and HPV-18 being the most common carcinogenic strains. Cervical cancer is preventable through primary (HPV vaccination) and secondary (Pap and HPV tests) prevention. These prevention methods should be promoted, however, in order to design a cancer prevention programme and the awareness and characteristics of populations should be investigated so that prevention programmes can be targeted specifically to them.

**Methods:**

In this work, we sought to investigate awareness of HPV and cervical cancer prevention among female healthcare workers in Cyprus. To achieve this, we conducted a 60-item survey among 200 healthcare professionals in randomly selected hospitals in two different cities within Cyprus.

**Results:**

Our results revealed that nearly 10% of our participants reported not ever having had a Pap test. 88.5% of the healthcare workers knew about HPV and 86.5% reported that HPV is transmitted through sexual intercourse. 83.5% of the participants were willing to vaccinate themselves for cervical cancer prevention.

**Conclusion:**

Even though awareness and vaccination acceptance were relatively high, they are still not optimal for healthcare professionals who play an essential role in health promotion. We suggest the design of educational programmes to target this population and improve their knowledge so that they can promote cervical cancer prevention in their health practice.

## Introduction

Globally, cervical cancer is the fourth most common cancer in women with 530,000 new cases diagnosed and more than 270,000 deaths every year [1]. The incidence rate of cervical cancer varies by geographic regions and age. Women aged 20–29 years have an incidence rate of 3.2 per 100,000 and women aged 40–49 years have an incidence of 14.2 per 100,000. However, the occurrence rate of the disease declines progressively after the age of 50 [2]. The age-adjusted incidence rate of cervical cancer in Cyprus has been stable for more than a decade (1998–2008) with a rate of 4.76 per 100,000 women for the latest period (2006–2008) [3]. In the latest reported year (2013) by the Ministry of Health, cervical cancer incidence actually rose to 6.4 per 100,000 [4]. A recent study of women in Cyprus with cytological abnormalities in the cervix has shown that 59.4% of those women were infected with high-risk HPV, 34.8% with low-risk HPV and 5.8% with HPV of unknown risk. Among the high-risk HPV types, the most common was HPV16 (17.7%), followed by HPV31 (12.9%), HPV58 (7.1%), HPV68 (4.6%), HPV18 (4.1%) and HPV56 (3.7%) [5]. When it comes to cervical cancer screening, only 67.4% of all women aged 20–69 are screened for cervical cancer every 3 years in Cyprus, with the largest proportion of women getting screened between 45 and 54 years old [6]. There are no screening guidelines for cervical cancer in Cyprus, where opportunistic screening occurs depending on the doctor’s recommendation.

The symptomology of the disease varies from having no symptoms to abnormal vaginal bleedings, unfamiliar vaginal discharges and pain during sexual intercourse [7]. A cornerstone in understanding the pathogenesis of cervical cancer was the identification of HPV as an aetiological agent. Persistent infections by HPV-16 and HPV-18 serotypes are the commonest cause of cervical cancer [8]. HPV 16 and 18 can also infect other organs and cause genital warts, oropharyngeal dysplasia and malignancies of penis, vagina and vulva [9]. Human papilloma virus is transmitted sexually. Hence, sexual activities and behaviour are the chief determinants of virus transmission. Age at first intercourse, having multiple sex partners and unprotected sex have been associated with the risk of HPV infection [10]. Epidemiological studies have also shown that low socioeconomic status, low educational level, use of contraceptive pills and being a current smoker increase the risk of HPV infection [11].

Cervical cancer is preventable. The availability of a cervical cytology test (Papanicolaou test also known as Pap test) and more recently HPV test has contributed to a decline in cervical cancer mortality. Furthermore, the development of a vaccine against various HPV strains is expected to further reduce cervical cancer incidence and mortality [12, 13]. There are three types of HPV vaccines: a bivalent vaccine against strains 16 and 18, a quadrivalent against strains 16, 18, 6 and 11 and a nonavalent against strains 16, 18, 31, 33, 45, 52, 58, 6 and 11 [14].

Even though vaccines against HPV have been shown to be effective in reducing the incidence of cervical cancer, awareness and knowledge about them still remains low. A recent study carried out in Nigeria showed that only 17.7% of the students had heard about human papilloma virus and only 14.4% were aware that HPV vaccines exist. Of the students who had heard of the HPV vaccine, only 46.2% knew that it was given to prevent cervical cancer [15]. In addition, a study of 1,476 individuals in Puerto Rico showed that only 37.2% of the participants were aware of HPV and 33.4% knew about the vaccine [16]. Another study on 1,578 mothers in Shandong, China showed that only 19.32% knew about HPV. Lack of knowledge about the virus was more common in women with low educational level and income [17].

Even more concerning is the lack of awareness and knowledge of HPV among healthcare providers who play a critical role in health promotion. A study in Singapore among 2,000 nurses reported that 81.9% of the nurses knew about the association of cervical cancer with multiple sexual partners, 78.2% knew about the association of cervical cancer with history of sexually transmitted diseases and 73.5% knew about the association of cervical cancer with HPV infection. However, the study revealed that the nurses had misconceptions about the efficacy, safety and indications of the vaccine. Only 54.6% of these nurses were aware of the preventive nature of the HPV vaccine and 20.5% had the misconception that the HPV vaccine is only indicated for women at high risk for cervical cancer [18]. A similar study in Turkey conducted in 2013 among 464 university hospital nurses revealed that none had received the HPV vaccine, only 13.8% had undergone cervical cancer screening and 11.6% had done the Pap smear test. 44% reported that they would not recommend the HPV vaccine to their patients due to their lack of awareness [19]. A study among 150 Greek female healthcare workers revealed that only 80% of surveyed women knew about the existence of HPV testing [20].

Considering the misconceptions surrounding HPV and cervical cancer prevention in various populations, we assessed awareness of HPV and cervical cancer prevention in female healthcare workers in Cyprus. In addition, we investigated their attitudes towards HPV vaccination. Healthcare workers are at the frontline of health promotion and hence it is crucial that they are aware of various ways of cervical cancer prevention, especially with the availability of primary prevention. We hope our results can guide the design of cervical cancer prevention education programmes for healthcare professionals that would ultimately result in better education of the general public regarding this issue.

## Materials and methods

### Study design

A cross-sectional survey was conducted between January and May 2015. Female healthcare workers were selected from hospitals in the cities of Nicosia and Larnaca based on cluster sampling. The hospitals were randomly selected from a list of hospitals in the two cities. The hospitals in Nicosia include one public hospital and seven private hospitals/clinics. The hospitals in Larnaca include one public and two private hospitals/clinics. The private hospitals/clinics in the two cities were randomly selected from a total of 27 and 13 private hospitals in the cities of Nicosia and Larnaca, respectively. Due to privacy and confidentiality laws, we performed convenience sampling within the hospitals, whereby the hospitals indicated the number of questionnaires they wanted us to leave them and they distributed them to their staff. The survey was anonymous and was distributed to 300 participants (self-administered) using a validated questionnaire with 60 questions in the Greek language. We distributed the survey to 300 participants assuming approximately 70% response rate. Two hundred participants completed and returned the questionnaire after informed consent. Sample size of 200 was estimated using the formula *N* = Z^2^ PQ/D^2^; where *N* = required sample size, Z = 1.96 at confidence level of 95%, P = 0.15 = estimated proportion of women not adhering to cervical cancer screening guidelines from a similar study we conducted in Greece, D = margin of error at 5% (standard deviation of 0.05) and Q = 1–P [20]. The study was conducted after approval by the Cyprus National Bioethics Committee (Case Number: ΕΕΒΚ ΕΠ 2015.01.22) and the University of Nicosia Bioethics Committee.

### Assessment tool

The questionnaire consisted of 60 questions that were distributed among four sections: 1) demographics (such as age, educational level, marital status and number of children), 2) general and gynaecological health behaviour (smoking, use of contraception, frequency of visits of the gynaecologist, frequency of pap tests, ever had abnormal cervical cytology and ever had an HPV test), 3) knowledge and awareness about HPV, cervical cancer and Pap test (risk factors of cervical cancer, means of cervical cancer prevention, prevalence of cervical cancer and knowledge about HPV, HPV transmission, HPV test and sources of knowledge) and 4) acceptance of HPV vaccination (for self, son and daughter and reasons for not accepting). The questionnaire was previously used in a study in Greece [21]. Permission for using the questionnaire in this study was obtained from the owner.

### Data analysis

The data were analysed using IBM SPSS statistical package version 20 using a coding system for all questions. Chi-Square test was used to determine whether differences among groups were significant. Multiple logistic regression was used (SAS 9.3 software) to generate models in order to study the association of multiple factors (age, education, marital status, smoking status, knowledge of cervical cancer prevention through protected sex or pap test screening) with different outcomes such as frequency of cervical cancer screening through Pap test and acceptance of HPV vaccination. The threshold for statistical significant was set at 0.05.

## Results

### Demographics

There was a wide distribution of age groups with 35% of the women at 20–29 years of age, 34% at 30–39 years of age, 15.5% at 40–49 years of age, 13.5% at 50–59 years of age and 1% older than 60 years (Table 1). As far as marital status is concerned, 31% of the participating women were single, 53% were married, 9% were divorced, 1.5% separated and 4.5% widowed (Table 1). The majority of the participants (88.5%) had higher (tertiary) education and 8% had high school education. Nurses comprised the largest proportion of the participants with a percentage of 73% followed by doctors 7.5% (Table 1).

### Cervical cancer screening behaviour

The percentage of participants that reported never having had a Pap test was 9.5. The main reasons recorded for not having the Pap test were negligence (42.1%), not experiencing symptoms (21%) and fear of the results (15.7%). Among the group that have done the Pap test, a significantly higher proportion of women (99%) older than 30 years had done the Pap test compared to women ages 20–29 years (74%) [*p* < 0.0001; df = 1; Chi-square statistic (with Yates correction) = 26.801; Chi-square test]. Almost three quarters (73%) of women reported having had Pap tests regularly for the past 5 years with a higher proportion of women over 30 years old (84%) compared to women ages 20–29 years (54%) [*p* < 0.001; df = 1; Chi-square statistic (with Yates correction) = 15.038; Chi-square test]. Multiple logistic regression revealed the positive association of cervical cancer screening frequency with age (older women had more frequent pap tests compared to younger women) and marital status (married women had more frequent pap tests compared to single women) (Table 2). Cervical cancer screening behaviour was not associated with education, knowledge of cervical cancer prevention or knowledge of Pap test. In addition, only 3% of the participants reported having had a positive Pap test and 37.5% reported knowing a patient or a friend who has had a positive Pap test.

### General health behaviour

We assessed general features of the health behaviour of the participants such as smoking, use of contraceptive pills and visits to the gynaecologist. The majority (81%) of participants reported being non-smokers and 16.5% reported being smokers. Among the smokers, 3% smoked for less than a year, 6.5% for 1–4 years, 3.5% for 5–10 years and 5% for more than 10 years. The percentage of women that reported visiting their gynaecologist once a year was 76.3%, while 9.8% reported never visiting the gynaecologist. The percentage of the participant women that reported not using contraceptive pills was 67. We found that a higher proportion of women taking oral contraceptive pills have done a Pap test (98.3%) compared to women not taking contraceptive pills (86.5%) (*p* = 0.0185).

### Awareness of human papilloma virus and cervical cancer

A large percentage (88.5%) of the participants reported that they know about the human papilloma virus and 86.5% selected sexual intercourse as a route of transmission of HPV (Table 3; multiple responses allowed). Nearly all (97%) of the participants reported that cervical cancer can be prevented and 80% reported that cervical cancer can be cured. Interestingly, 80.5% of participants indicated Pap test as a means of cervical cancer prevention, 51% considered conservative sexual relations as a means for cervical cancer prevention and 44% considered frequent check-up by gynaecologist as a means for cervical cancer prevention (Table 4; multiple responses allowed). Some participants felt that medical doctors were not adequately informed on the topic. More specifically, 11% and 17% of healthcare workers believed that gynaecologists and paediatricians, respectively, are not adequately informed about HPV and cervical cancer.

### Awareness and acceptance of HPV vaccine

Nearly one-fifth (18.33%) of healthcare workers reported that they are not aware of the existence of an HPV vaccine. The questionnaire also included questions about the participants’ acceptance of the vaccine for themselves, their daughters and their sons. Interestingly, 79.47% of the participants would accept getting vaccinated to protect themselves from HPV infection and 83.5% would accept vaccination to prevent cervical cancer. The major reason reported for refusing to get vaccinated was ‘fear of side effects’ (55.12% participants chose that answer) (Table 5). Acceptance to vaccinate themselves was negatively associated with age, and positively associated with the frequency of Pap tests and knowledge about cervical cancer prevention through Pap tests (Table 6). When participants were asked about vaccinating their daughters and sons to prevent them from HPV infection and genital warts, respectively, 82.87% responded positively for vaccinating their daughters and 79.09% responded positively for vaccinating their sons. Furthermore, 80% reported accepting to vaccinate their sons to prevent their future partners from HPV infection. There was some variation in the participants’ responses regarding whom they trust to vaccinate their daughters. More specifically, 39.36% of the participants reported trusting only a gynaecologist and 39.89% only paediatricians. When financial barriers for HPV vaccination were addressed, 71.5% of the participants reported they would get the vaccine even if it was not offered for free and 78.5% believed that the cost of the vaccine could be a potential reason for people not to get it.

## Discussion

Overall our study participants appeared to be attentive to their health as evidenced by the majority of them being non-smokers (81%) and 76.3% of them visiting their gynaecologists once a year. In addition, a high percentage of female healthcare workers have had the Pap test (88%). This number is similar to the 87.6% of women in the general population in Greece that reported to have had a Pap test [21]. However, our participants were healthcare professionals and therefore we expected a higher adherence to cervical cancer screening. Older age and being married were associated with having frequent Pap tests. This may be because older and married women have more frequent visits to gynaecologists due to pregnancies or the need for contraception and, therefore, there is more opportunity for discussion regarding the Pap test with their doctor. We had found similar results with regard to age and marital status association with having more frequent Pap tests in another study we had conducted among Greek female healthcare workers [20]. Nearly 10% of our participants reported not ever having had a Pap test with the main reasons being negligence, not experiencing symptoms and fear of results. This is worrisome since cervical cancer is often asymptomatic. Interestingly, the reasons for not having the Pap test are quite different from other neighbouring countries with different cultures and beliefs. For example, in Turkey, 28.2% of the women reported not having Pap tests because they are not sexually active, 18.3% do not consider themselves at risk and 16.8% are ashamed to do the test [19]. The knowledge about human papilloma virus among Cypriot healthcare workers was high, albeit with some gaps. More specifically, 88.5% of the healthcare workers knew about HPV and 86.5% reported that HPV is transmitted through sexual intercourse, leaving more than 10% of these healthcare workers unaware of HPV and its mode of transmission. However, these numbers are in sharp contrast to developing countries like India where only 18% of the nurses knew about HPV [22] and only young nurses were aware that HPV is transmitted sexually [23]. Interestingly, in our study, a small minority of the participants believed that HPV can be transmitted from sharing toilet seats or that the virus naturally exists in our bodies and causes cervical cancer. 86.8% of our study participants correctly noted that HPV is a causative agent of cervical cancer.

Results in different countries vary. For example, in a tertiary care hospital in Pakistan, only 61% of the nursing staff knew that HPV infection might lead to cervical cancer [24]. However, at Lagos University Teaching Hospital in Nigeria, 92% of the nurses knew about the link between HPV and cervical cancer. This may reflect differences in the types of hospitals. For example, Lagos University Teaching Hospital in Nigeria is a university hospital where continuing medical education is provided [25]. Interestingly, a similar study in Greece (which is a neighbouring country to Cyprus) among women of the general population revealed that only 43.2% of the women knew that the virus is sexually transmitted. This provides an opportunity for healthcare workers who are better informed to educate their patients on HPV and cervical cancer.

97% of our study participants were aware that cervical cancer is preventable. 10.5% reported avoiding smoking, 51% reported conservative sexual relations, 44% reported frequent check-up by gynaecologist and 80.5% reported the Pap test as important for cervical cancer prevention. This is different from a similar study we conducted among Greek female healthcare workers where 81.3% reported the Pap test but only 6.6% reported conservative sexual relations and 10% reported frequent check-up by gynaecologist [20]. Thus, while healthcare professionals in Cyprus recognise the importance of the Pap test, many of them rely on invalid preventive measures such as conservative sexual relations and frequent check-up by gynaecologist. Furthermore, some still classified incorrect or unproven measures such as healthy diet (5.5%) and even some medications and vitamin supplements (6.5%) as means of cervical cancer prevention. Regarding the Pap test, our results are also similar to a study in Turkey, where 75.2% of the nurses reported Pap test as a major means for preventing cervical cancer [23].

Only 81.67% of the female healthcare workers in our study knew about the HPV vaccine. However, awareness in Cyprus was better compared to other countries such as India where only 18% of nurses in a rural area knew about the HPV vaccine [22]. The acceptance of the vaccine was relatively high among our participants. 79.47% were willing to get vaccinated in order to protect themselves from HPV infection and 83.5% to protect themselves from cervical cancer. A possible explanation for this slight discrepancy could be a lack of knowledge that HPV infection leads to cervical cancer or the fear of suffering from cancer is greater than that of viral infection. Younger women were more accepting to vaccinate themselves, and so were women who had frequent Pap tests and knew about cervical cancer prevention through Pap tests. This suggests that education regarding cervical cancer prevention would likely improve vaccination acceptance. Among those who refused to get the HPV vaccine, 55.12% reported fear of the side-effects as the main reason, 30.60% reported that they are not sufficiently informed about the vaccine and 14.28% do not believe that the vaccine has a protective effect against infection. Education programmes should specifically address these fears and misconceptions.

This study has some limitations as well. We sampled only from hospitals in two of the five districts in Cyprus. Knowledge and screening behaviour may be different in the other districts. Due to privacy and confidentiality laws, there was no random selection of healthcare professionals from each hospital/clinic but rather everyone was invited to answer the questionnaire by the hospital administration. It may be that those who answered the questionnaire are more attentive to their health than those who did not. Another limitation is that we included in our analysis questionnaires with some missing questions because we felt by excluding them we may be creating other biases.

## Conclusions

Our study established that there is a high percentage of female healthcare workers who undergo cervical cancer screening in Cyprus. However, there were some misconceptions about HPV transmission and preventive measures for cervical cancer. This might be due to the fact that this group has access to gynaecologists and hence are advised to do the Pap test; however, they do not necessarily know details about cervical cancer prevention. These misconceptions should be addressed since healthcare workers are health promotion ambassadors. Finally, the reluctance surrounding HPV vaccination should be addressed by providing further vaccine-related education to healthcare professionals so that they will feel more inclined to promote it to their patients and thus increase HPV vaccination rates in their communities.

## Funding

This research did not receive any specific grant from funding agencies in the public, commercial or not-for-profit sectors.

## Conflicts of interest

The authors declare that they have no conflicts of interest.

## Figures and Tables

**Table 1.** Demographics and characteristics of the participants.

Age	Number (%)
20–29	70 (35)
30–39	68 (34)
40–49	31(15.5)
50–59	27 (13.5)
>60	2 (1)
N/A	2 (1)
Total	200
**Education level**	
Lyceum (high school diploma)	16 (8)
University level	78 (39)
Technical University	48 (24)
Post-graduate level	49 (24.5)
Ph.D.	2 (1)
N/A	7 (3.5)
Total	200
**Occupation**	
Doctor	15 (7.5)
Nurse	146 (73)
Health visitor	5 (2.5)
Midwife	10 (5)
Social worker	3 (1.5)
Nurse assistant	5 (2.5)
Ward assistant	10 (5)
Other	6 (3)
Total	200
**Marital status**	
Single	62 (31)
Married	106 (53)
Divorced	18 (9)
Separated	3 (1.5)
Widowed	9 (4.5)
N/A	2 (1)
Total	200

**Table 2.** Factors associated with more frequent cervical cancer screening by Pap test.

Variable	Odds ratio	95% CI
Age	0.265	0.129, 0.544
Education	1.189	0.562, 2.516
Marital status	0.762	0.27,2 2.139
Smoking	0.030	0.003, 0.278
Knowledge of cervical cancer prevention (protected sexual intercourse)	0.083	0.018, 0.390
Knowledge of cervical cancer prevention (Pap test)	0.872	0.166, 4.581
		Model *p* < 0.0001

**Table 3.** Knowledge about the transmission of human papilloma virus.

	*N* (%)
Sexual partner	173 (86.5)
Toilet seat	2 (1)
The virus simply exists in the body	5 (2.5)
Other	2 (1)
I don’t know	6 (3)
Total participants	200 (100)

**Table 4.** Knowledge about the prevention of cervical cancer.

Healthy diet	*N* (%)
Avoid smoking	11 (5.5)
Conservative sexual relations	21 (10.5)
Pap test	102 (51)
Frequent check-up by gynaecologist	161 (80.5)
Medication and vitamin supplements	88 (44)
Other	13 (6.5)
I don’t know	4 (2)
Total participants	200 (100)

**Table 5.** Reasons for refusing HPV vaccination.

	*N* (%)
Not sufficiently informed	15 (30.60%)
Afraid of the side effects of the drug	27 (55.12%)
Do not believe that the vaccine will prevent the infection	7 (14.28%)
Total	49(100%)

**Table 6.** Acceptance of HPV vaccination for themselves.

Variable	β-Coefficient	*p*-Value
Age	−0.114	0.0012
Marital status	0.068	0.090
Smoking	0.026	0.736
Frequency of Pap test	0.090	0.039
Knowledge of cervical cancer prevention (protected sexual intercourse)	0.047	0.119
Knowledge of cervical cancer prevention (Pap test)	0.233	0.0047
		Model *p*-value = 0.0002 *R*^2^ = 0.1581

## References

[1] Jemal A (2011). Global cancer statistics. CA Cancer J Clin.

[2] CDC (2012). Human papillomavirus-associated cancers-United States, 2004–2008, in Morbidity and Mortality Weekly Report. Centers for Disease Control and Prevention.

[3] Cooter M (2015). Incidence and time trends of cancer in Cyprus over 11 years (1998–2008). Tumori.

[4] Pavlou P, Demetriou A (2016). Progress Report. Cyprus Cancer Registry.

[5] Krashias G, Koptides D, Christodoulou C (2017). HPV prevalence and type distribution in Cypriot women with cervical cytological abnormalities. BMC Infect Dis.

[6] Bruni L ICO/IARC information centre on HPV and cancer (HPV information centre). Human papillomavirus and related diseases in Cyprus Summary Report 27 July 2017 [Date Accessed].

[7] American Cancer Society (2016). Signs and symptoms of cervical cancer. http://www.cancer.org/cancer/cervicalcancer/detailedguide/cervical-cancer-signs-symptoms.

[8] Munoz N (2003). Epidemiologic classification of human papillomavirus types associated with cervical cancer. N Engl J Med.

[9] Viens LJ (2016). Human papillomavirus-associated cancers—United States, 2008–2012. MMWR Morb Mortal Wkly Rep.

[10] Ribeiro AA (2015). HPV infection and cervical neoplasia: associated risk factors. Infect Agent Cancer.

[11] Cotton SC (2007). Lifestyle and socio-demographic factors associated with high-risk HPV infection in UK women. Br J Cancer.

[12] Saslow D (2007). American Cancer Society Guideline for human papillomavirus (HPV) vaccine use to prevent cervical cancer and its precursors. CA Cancer J Clin.

[13] Cutts FT (2007). Human papillomavirus and HPV vaccines: a review. Bull.

[14] MerckVaccines.com.

[15] Makwe CC, Anorlu RI, Odeyemi KA (2012). Human papillomavirus (HPV) infection and vaccines: knowledge, attitude and perception among female students at the University of Lagos, Lagos, Nigeria. J Epidemiol Glob Health.

[16] Reyes JC (2015). Demographic and high-risk behaviors associated with HPV and HPV vaccine awareness among persons aged 15–74 years in Puerto Rico. P R Health Sci J.

[17] Yu Y (2016). Human papillomavirus infection and vaccination: awareness and knowledge of hpv and acceptability of hpv vaccine among mothers of teenage daughters in Weihai, Shandong, China. PLoS One.

[18] Tay SK (2015). Vaccine misconceptions and low hpv vaccination take-up rates in Singapore. Asian Pac J Cancer Prev.

[19] Koc Z, Cinarli T (2015). Cervical cancer, human papillomavirus, and vaccination: knowledge, awareness, and practices among Turkish Hospital Nurses. Nurs Res.

[20] Farazi PA, Hadji P, Roupa Z (2017). Awareness of human papilloma virus and cervical cancer prevention among Greek female healthcare workers. Eur J Cancer Prev.

[21] Agorastos T (2014). Epidemiology of HPV infection and current status of cervical cancer prevention in Greece: final results of the LYSISTRATA cross-sectional study. Eur J Cancer Prev.

[22] Singh E (2012). Awareness of cervical cancer screening among nursing staff in a tertiary institution of rural India. J Gynecol Oncol.

[23] Yaren A (2008). Awareness of breast and cervical cancer risk factors and screening behaviours among nurses in rural region of Turkey. Eur J Cancer Care (Engl).

[24] Ali SF (2010). Knowledge and awareness about cervical cancer and its prevention amongst interns and nursing staff in Tertiary Care Hospitals in Karachi, Pakistan. PLoS One.

[25] Awodele O (2011). A study on cervical cancer screening amongst nurses in Lagos University Teaching Hospital, Lagos, Nigeria. J Cancer Educ.

